# Magnetic resonance assessment of left ventricular volumes and mass using a single-breath-hold 3D k-t BLAST cine b-SSFP in comparison with multiple-breath-hold 2D cine b-SSFP

**DOI:** 10.1007/s13244-010-0056-1

**Published:** 2010-12-18

**Authors:** Erica Maffei, Giancarlo Messalli, Chiara Martini, Alexia Rossi, Niels van Pelt, Robert-Jan van Geuns, Annick C. Weustink, Nico R. Mollet, Koen Nieman, Annachiara Aldrovandi, Massimo Imbriaco, Jan Bogaert, Filippo Cademartiri

**Affiliations:** 1 Department of Radiology and Cardiology, University Hospital, Parma, Italy; 2 Department of Radiology, SDN Foundation IRCCS, Naples, Italy; 3 Department of Radiology and Cardiology, Erasmus Medical Center, Rotterdam, The Netherlands; 4 Department of Biomorphological and Functional Sciences, University Federico II, Naples, Italy; 5 Department of Radiology, Gasthuisberg University Hospital, Catholic University of Leuven, Leuven, Belgium; 6 Department of Radiology, c/o Piastra Tecnica—Piano 0, Azienda Ospedaliero-Universitaria, Via Gramsci, 14, 43100 Parma, Italy

**Keywords:** Magnetic resonance imaging, Cardiac MRI, 2D b-SSFP, 3D b-SSFP, k-t BLAST, Left ventricle, Volumetric quantification, Ejection fraction

## Abstract

**Objective:**

To assess the feasibility of single-breath-hold three-dimensional cine b-SSFP (balanced steady-state free precession gradient echo) sequence (3D-cine), accelerated with k-t BLAST (broad-use linear acquisition speed-up technique), compared with multiple-breath-hold 2D cine b-SSFP (2D-cine) sequence for assessment of left ventricular (LV) function.

**Methods:**

Imaging was performed using 1.5-T MRI (Achieva, Philips, The Netherlands) in 46 patients with different cardiac diseases. Global functional parameters, LV mass, imaging time and reporting time were evaluated and compared in each patient.

**Results:**

Functional parameters and mass were significantly different in the two sequences [3D end-diastolic volume (EDV) = 129 ± 44 ml vs 2D EDV = 134 ± 49 ml; 3D end-systolic volume (ESV) = 77 ± 44 ml vs 2D ESV = 73 ± 50 ml; 3D ejection fraction (EF) = 43 ± 15% vs 2D EF = 48 ± 15%; *p* < 0.05], although an excellent correlation was found for LV EF (*r* = 0.99). Bland-Altman analysis showed small confidence intervals with no interactions on volumes (EF limits of agreement = 2.7; 7.6; mean bias 5%). Imaging time was significantly lower for 3D-cine sequence (18 ± 1 s vs 95 ± 23 s; *p* < 0.05), although reporting time was significantly longer for the 3D-cine sequence (29 ± 7 min vs 8 ± 3 min; *p* < 0.05).

**Conclusions:**

A 3D-cine sequence can be advocated as an alternative to 2D-cine sequence for LV EF assessment in patients for whom shorter imaging time is desirable.

## Introduction

Significant left ventricular (LV) dysfunction is associated with poor prognosis. The reliable determination of LV function is an important component of the cardiac evaluation in several clinical settings [1–3].

Previous studies demonstrated that magnetic resonance imaging (MRI) is an accurate and reproducible technique for the measurement of LV volumes and is currently regarded as the reference standard [4–6]. A two-dimensional (2D) balanced steady-state free precession gradient echo sequence (b-SSFP) is currently considered the preferred method to assess LV volume and function because of its high spatial and temporal resolution [5, 7–9]. However, 2D b-SSFP sequence has an important limitation in that it requires multiple prolonged breath-holds that increase examination time and may cause patient restlessness and slice mis-registration [10, 11]. Recently, a new speed-up technique, k-t BLAST (broad-use linear acquisition speed-up technique), has become available; it allows an undersampling in the temporal domain and, applied to a 3D-cine b-SSFP sequence, may allow coverage of the entire left ventricle in a single breath-hold, as explained in previous papers [11–14]. The performance of this 3D-cine sequence has until now not been evaluated in a clinical setting [15, 16]. Furthermore, most authors used two breath-holds to have the volume dataset acquired available for analysis; they performed the second breath-hold to acquire the “training dataset” [16, 17].

We set a true single breath-hold 3D sequence, with no necessity to acquire the “training data” with a second breath-hold, and we sought to investigate the variability of LV volume and mass measurements and the time efficiency of this new single breath-hold 3D-cine sequence using a standard multiple breath-hold 2D-cine sequence as reference.

## Materials and methods

### Patient population

Forty-eight patients, who were referred for MRI assessment of LV function for different indications, were prospectively enrolled for the study. Exclusion criteria for MRI were the standard absolute and relative contraindications for MRI: patients with claustrophobia, pace-maker and other MRI-incompatible devices were not considered for this study [18]. The inclusion criterion was the ability to perform a breath-hold of at least 18 s. The study was approved by the local Ethics Committee and all patients gave informed consent.

### MRI data

All imaging was performed in the Tertiary Referral Hospital (Parma, Italy), using a 1.5-T MRI (Achieva, Philips Medical Systems, Best, The Netherlands) with: maximum gradient strength 66 mT/m, maximum slew rate 180 mT/m × ms, maximum gradient strength during cine-cardiac MRI acquisition 33 mT/m, and maximum slew rate during cine-cardiac MRI acquisition 180 mT/m × ms. Five-element synergy cardiac coil and vector electrocardiography were used for signal detection and cardiac gating. One experienced operator (3 years’ cardiac MRI) performed all the examinations. After initial scout imaging and reference acquisition, both the 2D b-SSFP (2D-cine) and the 3D b-SSFP k-t BLAST (3D-cine) sequences were acquired on the short axis plane covering the entire left ventricle. The imaging parameters for 2D and 3D sequences are listed in Table 1.

**Table 1:** Sequence parameters

	TR	TE	Flip angle	Bandwidth	In plane resolution	Slice thickness	Slice gap	Temporal resolution	Cardiac phase	SENSE	Partial image	K-t BLAST factor
2D-cine	3.1	1.53	60º	1,249.7 Hz/pixel	2 × 2.3 mm	8 mm	2 mm	32 ± 6 ms	30	Off	Yes	0
3D-cine	3.2	1.59	50º	1,388.5 Hz/pixel	2.4 × 2.7	5 mm	0	59 ± 11 ms	16	Off	Yes	4

*TR* time of repetition, *TE* time of echo

We used a retrospective gated sequence for 2D-cine and a gated sequence for 3D-cine because software constraints did not permit a true retrospective gating.

### Data analysis

Two experienced observers (3 and 4 years’ cardiac MRI) analysed images with an off-line post-processing workstation (ViewForum release 4.2, Philips Medical System). First, a visual evaluation was performed on each volume dataset distinguishing reportable and non-reportable datasets. Next, the end-diastolic and end-systolic phases were identified; the first image of the cardiac cycle (0%) was considered to be end-diastolic, whereas the image with the smallest LV cavity was considered to be end-systolic; then both the endocardial and epicardial contours were manually traced on the short-axis views on end-diastolic phase images, endocardial borders were “propagated” thanks to the software on end-systolic phase images and finally manual correction of contours was performed by the operators. On end-diastolic and end-systolic phase images, the first section of the LV with a visible lumen was defined as the apex, whereas the base was defined as the most basal section surrounded by at least 50% myocardium. Papillary muscles and trabeculations in the LV cavity were included in the LV volume as previously described [19]. One observer analysed images a second time to assess the intra-observer variability.

**Fig. 1a–d:**
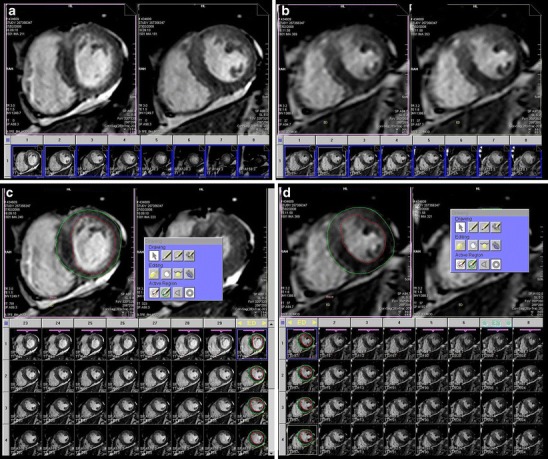
Examples of 2D and 3D sequences. The figure shows the same patient studied with the two different sequences. In **a** and **b** the 2D- and 3D-sequences are displayed in the ARGUS (Siemens, Germany) platform before the quantitative assessment. In **c** and **d**, the 2D and 3D sequence are displayed during the contour detection in the end-diastolic phase, respectively

Cardiac functional parameters were evaluated in both 2D-cine and 3D-cine sequences (Fig. 1). In particular, end-diastolic volume (EDV), end-systolic volume (ESV), LV ejection fraction (EF), stroke volume (SV) and end-diastolic wall mass of the left ventricle (ED wall mass). We reported the acquisition time and the evaluation time for each patient and for each sequence. Acquisition time for 2D cine multiple breath-holds was reported as “effective acquisition time”, where the effective acquisition time was calculated taking into account the “recovery time” between the single breath-holds. We also calculated the temporal resolution obtained with 2D-cine and 3D-cine sequences for each patient: temporal resolution was calculated by dividing the R-R interval for the number of phases inherent to each type of sequence.

### Statistical analysis

The 2D-cine sequence was used as the reference standard. The performance of 2D-cine and 3D-cine were compared using the paired Student’s *t*-test and a *p* < 0.05 was defined as statistically significant. Correlations between EDV, ESV, LV EF, and LV ED wall mass between the two sequences were assessed using Pearson’s correlation analysis. Agreement on the LV parameters between 2D and 3D sequences were assessed using Bland-Altman analysis.

The time-efficiency differences were made using the paired Student’s *t*-test and a *p* < 0.05 was defined as statistically significant.

Statistical analysis exploring the impact of intra- and inter-observer variability was performed using the same methodology.

## Results

**Fig. 2:**
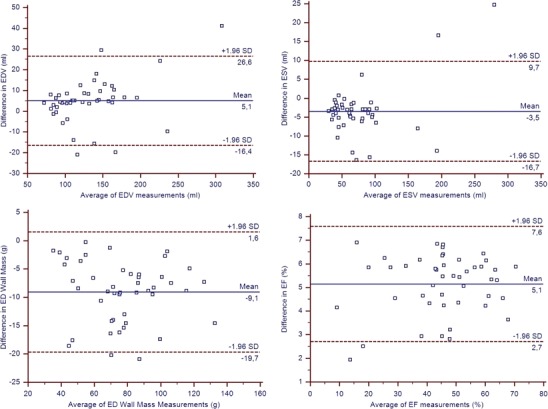
Bland-Altman plots. A good agreement was found for EDV and ESV; a dispersion of measurement was found for the EF and the ED wall mass

Of the enrolled population (48 patients) undergoing 2D-cine and 3D-cine sequences, 46 patients had visually optimal image quality, and we had to exclude two patients because of blurring artefacts. The population’s characteristics are summarised in Table 2. All calculated parameters with the two sequences showed significant differences (*p* < 0.05). In particular, we observed an under-estimation of EDV (129 ± 44 ml vs 134 ± 49 ml, *p* < 0.05) and over-estimation of the ESV (77 ± 44 ml vs 73 ± 50 ml, *p* < 0.05) in 3D-cine. This resulted in an under-estimation of EF (43 ± 15% vs 48 ± 15% with *p* < 0.01), SV (52 ± 19 ml vs 60 ± 21 ml with *p* < 0.05) and CO (3.3 ± 1.3 ml vs 3.9 ± 1.3 ml with *p* < 0.05) in 3D-cine. Despite the differences, an excellent correlation was found between measurements, in particular for EF (*r* = 0.99; Table 3). Bland-Altman analysis on LV functional parameters between 2D-cine and 3D-cine sequences, using limits of agreement (±1.96 SDs from the mean difference), showed good results. The mean difference for the EF was 5% (limits of agreement 2.7–7.6%; Fig. 2).

**Table 2:** Baseline characteristics

Parameters	Values
Number of patients	46
Male/Female	29/17
Mean age (years) ± SD	51 ± 19
Mean weight (kg) ± SD	75 ± 16
Mean heart rate (bpm) ± SD	65 ± 13
Clinical indications	Values
CAD	29
DCM	9
HCM	3
Valve study	3
ARVD	2

*SD* standard deviation, *CAD* coronary artery disease, *DCM* dilated cardiomyopathy, *HCM* hypertrophic cardiomyopathy, *ARVD* arrhythmogenic right ventricular dysplasia

**Table 3:** Comparison of global LV parameters calculated with 2D and 3D k-t BLAST sequences. Parameters are expressed as mean ± SD; *p value* Student’s paired test; *r value* Pearson’s correlation; *95% LA* limits of agreement with Bland-Altman analysis

LV Parameters	2D	3D k-t BLAST	*p* value	*r* value	95% LA
EDV (ml)	134 ± 49	129 ± 44	<0.05	0.98	-16.4; 26.6
ESV (ml)	73 ± 50	77 ± 47	<0.05	0.99	-16.7; 9.7
SV (ml)	60 ± 21	52 ± 19	<0.05	0.96	-2.7; 19.9
EF (%)	48 ± 15	43 ± 15	<0.01	0.99	2.7; 7.6
CO (l/min)	3.9 ± 1.3	3.3 ± 1.3	<0.05	0.95	-0.20; 1.34
ED wall mass (g)	72 ± 23	81 ± 24	<0.05	0.97	-19.7; 1.6

*LV* left ventricle, *EDV* end diastolic volume, *ESV* end systolic volume, *SV* stroke volume, *EF* ejection fraction, *CO* cardiac output, *ED wall mass* end diastolic wall mass

Time-efficiency was calculated taking into account the effective acquisition time and reporting time. Total imaging time was significantly shorter for 3D-cine than for multiple 2D-cine (18 ± 1 s vs 95 ± 23 s with *p* < 0.05). The average heart rate was not significantly different during 3D-cine and 2D-cine sequences (65 ± 13 bpm vs 66 ± 13 bpm with *p* = 0.50) and sequence-related temporal resolution was significantly lower in 3D acquisition (59 ± 11 ms vs 32 ± 6 ms with *p* < 0.0001). However, reporting time was significantly longer for the 3D-cine sequence (29 ± 7 min vs 8 ± 3 min with *p* < 0.05). Intra- and inter-observer variability was within expected ranges (Table 4) [9, 20].

**Table 4:** Intra- and inter-observer variability and the comparison between 2D and 3D k-t BLAST in terms of global LV volume parameters and reporting time. Parameters are expressed as mean ± SD; *p value* Student’s paired test; *r value* Pearson’s correlation; *95% LA* limits of agreement with Bland-Altman analysis

	Intra-observer					Inter-observer				
3D k-t BLAST	1st analysis	2nd analysis	*p* value	*r* value	95% LA	1st observer	2nd observer	*p* value	*r* value	95% LA
EF (%)	43 ± 15	43 ± 15	>0.05	0.99	1.75; -2.23	43 ± 15	41 ± 14	<0.05	0.96	10.2; -6.5
ED wall mass (g)	81 ± 24	82 ± 24	>0.05	0.99	3.8; -4.1	81 ± 24	88 ± 24	<0.05	0.93	10.6; -24.3
Report time (min)	29 ± 7	29 ± 7	>0.05	0.99	2.5; -2.1	29 ± 7	32 ± 7	<0.05	0.89	3.1; -10.2
2D	1st analysis	2nd analysis				1st observer	2nd observer			
EF (%)	48 ± 15	48 ± 15	>0.05	0.99	2.3; -1.7	48 ± 15	46 ± 14	<0.05	0.96	9.8; -5.9
ED wall mass (g)	72 ± 23	72 ± 23	>0.05	0.99	6.8; -4.9	72 ± 23	76 ± 23	<0.05	0.92	13.9; -20.5
Report time (min)	8 ± 3	8 ± 2	>0.05	0.90	2.2; -2.7	8 ± 3	9 ± 3	<0.05	0.64	3.2; -6.4
2D vs 3D k-t BLAST	2D	3D k-t BLAST				2D	3D k-t BLAST			
	2nd analysis	2nd analysis				1st observer	2nd observer			
EF (%)	48 ± 15	43 ± 15	<0.05	0.99	6.56; 2.75	46 ± 14	41 ± 14	<0.05	0.99	7.9; 2.2
ED wall mass (g)	72 ± 23	82 ± 24	<0.05	0.98	0.4; -20.4	76 ± 23	88 ± 24	<0.05	0.96	-0.5; -24.7
Report time (min)	8 ± 2	29 ± 7	<0.05	0.25	-6.9; -34.4	9 ± 3	29 ± 7	<0.05	0.16	-4.8; -34.1

## Discussion

MRI 2D-cine b-SSFP sequences are considered the reference standard for the assessment of LV volumes and mass [21]. The idea of a single breath-hold MR sequence is appealing because it may reduce inaccuracies introduced by prolonged multiple breath-holds, such as slice mis-registration, but it requires speeding up image acquisition.

In cardiac imaging, acquisition speed has always been of primary importance; during the past few years several tricks have been used with this aim; in the beginning, speed improvement was achieved thanks to gradient hardware but a further improvement has been limited by physiological constraints such as peripheral nerve stimulation. Thanks to the parallel imaging (such as SENSE and SMASH), using information from multiple receiver coils, it has been possible to reconstruct images from a sparsely sampled set of data recovering the missed information by exploiting the differences in signals detected by multiple receiver coils [21]. In clinical practice with parallel imaging, a maximum acceleration factor of two can be achieved, and it is not possible to further increase it without decreasing the signal-to-noise ratio. To further improve the acquisition speed, undersampling techniques, operating in the temporal domain, have been developed, such as k-t BLAST, which can easily handle a fourfold acceleration factor. This new approach is based on the fact that image frames are only slightly different if acquired at different time points; by identifying the redundant information among the image frames, only the novel portions of the images need to be acquired, thus reducing the total amount of sampled information [21].

We evaluated a recently introduced volumetric sequence (i.e. 3D k-t BLAST) allowing single breath-hold functional assessment of the left ventricle.

Correlation and reproducibility of parameters were optimal. However, all measurements of the major cardiac indexes were significantly different (*p* < 0.05); differences may be explained as follows.

First, there are some intrinsic constraints in the software and hardware that do not permit the implementation of a true retrospective gating with 3D-cine sequence on commercially available systems, as extensively explained in a previous technical paper [16]. We think that even if our sequence parameters were optimally set, we may still not have been able to acquire the correct end-diastolic phase during acquisition and therefore it was not available for image analysis; this problem is highlighted by the constant underestimation of EDV assessed with the 3D-cine sequence compared with the 2D-cine sequence; this observation is in agreement with previous studies [14]. This observation may also explain the overestimation of the ED wall mass by the 3D-cine sequence. LV myocardium may have been evaluated when incompletely relaxed, producing an overestimation of ED wall mass.

Second, we found a constant overestimation of ESV with 3D-cine. The explanation may be related to the lower temporal resolution of 3D-cine, which reduces the accuracy in the selection of the proper end-systolic phase compared with the 2D-cine sequence; this is in agreement with previous studies exploring the impact of sequences with low temporal resolution [22].

Despite significant differences in the absolute values, the over- and under-estimation of LV volumes appeared systematic, resulting in a good correlation between functional parameters.

The most useful LV parameter is arguably the LV ejection fraction. The mean difference in the calculated EF between 3D-cine and 2D-cine was 5%, with relatively low limits of agreement. Our results suggests that the 3D-cine sequence may be used to obtain useful LV EF measurements in a single breath-hold in patients for whom shorter imaging time is desirable, taking into account both systematic bias and that follow-up studies should be performed with same technique. Anyway, in the future, implementation of a truly retrospectively gated 3D sequence is expected to overcome even the systematic bias encountered.

In order to perform a complete LV volumetric quantification, the 3D-cine sequence was more time consuming despite the shorter imaging time; this was because 3D-cine sequences (5-mm slice thickness and no gap) generate more slices to be analysed than 2D-cine sequences (8-mm slice thickness and 2-mm slice gap).

### Limitations

Three-dimensional sequence analysis could benefit from using a 3D analysis tool which permits the valvular and apex contours to be traced in long-axis views to better delineate endo- and epi-cardial borders at these levels; however, this tool was not yet available in our institute and therefore we did not use it.

## Conclusions

The parameters assessed in the 3D k-t BLAST cine b-SSFP sequence showed significant difference but excellent correlation and good agreement compared with the current 2D-cine sequence. This sequence may be an alternative technique for evaluating LV EF in patients for whom shorter imaging times are desirable.
